# Efficacy of Stent Thrombectomy Alone or Combined With Intermediate Catheter Aspiration for Severe Cerebral Venous Sinus Thrombosis: A Case-Series

**DOI:** 10.3389/fneur.2021.783380

**Published:** 2022-01-25

**Authors:** Tao Peng, Bitang Dan, Zini Zhang, Bifeng Zhu, Jianlin Liu

**Affiliations:** ^1^Department of Neurology, The Third People's Hospital of Hubei Province, Jianghan University, Wuhan, China; ^2^Department of Neurology, Zhongnan Hospital, Wuhan University, Wuhan, China

**Keywords:** stent thrombectomy, intermediate catheter aspiration, balloon dilation, anticoagulation treatment, severe cerebral venous sinus thrombosis stent thrombectomy, severe cerebral venous sinus thrombosis

## Abstract

**Objective:**

To assess the safety and efficacy of stent thrombectomy alone or combined with intermediate catheter aspiration for severe cerebral venous sinus thrombosis.

**Method:**

We retrospectively collected the clinical data of 7 patients with severe CVST who received endovascular treatment at our hospital from January 2017 to June 2020. The patients had at least one adverse prognostic factor (mental status disorder, comatose state, intracerebral hemorrhage, or thrombosis of the deep venous system).

**Results:**

The median age was 51 years. Three patients were women. Two or more venous sinuses were in involved in 5 patients. All patients received systemic anticoagulant therapy before endovascular therapy. Neurological deterioration was the main reason for patients with cerebral venous sinus thrombosis undergoing intravascular therapy. The median time from admission to intravascular therapy was 3 days (1–9 days). Primary endpoints: 6 patients (85.7%) had a modified Rankin scale score of 0 at day 90, and 1 patient (14.3%) had a modified Rankin scale score of 2 at day 90. Secondary endpoints: complete recanalization was achieved in 4 cases (57.1%) and partial recanalization in 3 cases (42.9%).

**Conclusion:**

Stent thrombectomy, combined with intermediate catheter aspiration, balloon dilation, and regional thrombolysis/anticoagulation treatment, is an effective strategy to treat severe cerebral venous sinus thrombosis patients who had inadequate response to anticoagulant therapy. This strategy can quickly eliminate the occluded venous sinus and improve prognosis of severe cerebral venous sinus thrombosis.

## Introduction

Cerebral venous sinus thrombosis (CVST) is a rare type of stroke. The incidence of CVST in adults is 1.32 per 100,000 persons per year (1). However, the incidence of CVST in women (aged 31–50 years) is 2.78 per 100,000 per year (2). Anticoagulant therapy is the first-line treatment for CVST (3), but nearly 20% of patients encounter disability or death (4). The baseline factors related to poor prognosis include disordered mental status, comatose state, intracerebral hemorrhage, or thrombosis of the deep venous system (4). Although there is no firm recommendation, the American Heart Association suggested that intravascular therapy be used as a remedial treatment for patients with inadequate response to anticoagulant therapy (5).

Endovascular therapy has been found to be effective against CVST, but relevant research is mostly single-center and retrospective (6–9). Two systematic studies (10, 11) on endovascular treatment for severe CVST and one randomized controlled trial (12) failed to find any advantage of endovascular therapy compared with anticoagulation therapy alone for patients with severe CVST. Nevertheless, studies have shown that endovascular treatment is safe and effective in patients with severe CVST. From these two systematic studies and one clinical trial, we found the following items: 1. Intrasinus thrombolysis was the most commonly used surgical modality. For example, there was an application rate of 87.8% in the study by Ilyas et al. (10), 71% in the study by Siddiqui et al. (11), and 52% in the TO-ACT study (12). 2. The Angiojet was the most commonly used device, with an application rate of 26% reported by Ilyas et al., 40% in the study by Siddiqui et al. research, and 42% in the TO-ACT study. Nevertheless, the Angiojet is a peripheral endovascular device with poor compliance, a high risk of vascular penetration, a low recanalization rate, and a low rate of a good outcome, which has long been mentioned (11). 3. The use of retrievable stents or aspiration systems was low. The rate of solitaire use was 11%, and penumbra was 9% according to Ilyas et al., penumbra was 7% in the study by Siddiqui et al., and retrievable stent use was 15% in the TO-ACT study. The new generation of retriever stents and aspiration devices can significantly improve clinical outcomes with acute cerebral infarction caused by large vessel occlusion. Still, few studies (13–15) have reported on the application of retriever stents and aspiration devices for the treatment of CVST. The purpose of this study was to assess the safety and efficacy of stent thrombectomy alone or combined with intermediate catheter aspiration for the treatment of severe CVST.

## Methods

### Patients

A retrospective review was performed of seven patients with severe CVST who underwent endovascular therapy between January 2017 and June 2020 at the Department of Neurology of the Third People's Hospital of Hubei Province.

The study was approved by the Ethics Committee of the Third People's Hospital of Hubei Province.

### Inclusion and Exclusion Criteria

The inclusion criteria for patients in our study were as follows: 1. Definite diagnosis of CVST based on head magnetic resonance imaging (MRI) of cerebral venous sinus vessels (MRV). 2. At least one risk factor highly suggestive of poor outcome (4), e.g., abnormal mental state, coma status (Glasgow Coma Scale score < 9), intracranial hemorrhage, and deep cerebral vein thrombosis. 3. After admission to hospital, clinical symptoms of patients continually deteriorated with systemic anticoagulant therapy (low molecular weight heparin 90–100 IU/kg/Q12 h), with an increase of ≥ 4 points in National Institute Health Stroke Scale (NIHSS) scores. 4. Neurocritical physicians and neuro-interventionalist decided to proceed with endovascular treatment. 5. Patient or a patient's family member signed informed consent.

Exclusion criteria for patients were as follows: 1. Time from diagnosis to endovascular treatment longer than 10 days; 2. pregnancy (puerperal women were appropriate); 3. thrombocytopenia (platelet count < 100 × 10 ^∧^ 9/L); 4. major surgery in the prior 2 weeks (lumbar puncture excluded); 5. clinical and radiographic evidence of supratentorial giant space-occupying signs; 6. other diseases with poor short-term prognosis not associated with CVST.

After the acute phase, all patients were required to be treated with a vitamin K antagonist. Patients who were using oral contraceptives received 3 months of anticoagulant treatment and patients with unexplained or mild inherited thrombophilia received 6–12 months of anticoagulant treatment. Patients with severe inherited thrombophilia received long-term anticoagulant treatment.

### Data Collection

The collected data included (baseline) age, sex, clinical presentation, etiology, time from diagnosis to endovascular treatment, and imaging characteristics. Operative details included surgical instruments, a strategy of thrombus removal, number of thrombi removed, and duration of surgery. The primary endpoint was modified Rankin scale (mRS) score at 90 days. The secondary endpoint was recanalization status, and procedural safety measures were the presence or absence of new intracranial hemorrhage (bleeding occurring within 1 week postoperatively that resulted in a ≥ 4-point increase in the patient's NIHSS assessment or death) or increased postoperative intracranial hematoma, hematoma at the puncture site, or vascular penetration.

### Technique

1. The left femoral artery was punctured, and a 5F femoral sheath was placed. A 5F VER catheter (Cordis Corp., USA) was established in the C1 segment of the internal carotid artery on one side for super-selective cerebral angiography to identify the site of venous sinus occlusion.
2. After puncture of the right femoral vein, an 8F femoral sheath was placed. Under the guidance of a loach guide wire (TERUMO, Vietnam), a 5F multifunctional catheter (125 cm Cordis Corp.) and a 6F Neuro Max long sheath (90 cm Penumbra, Inc., USA) were placed in the distal segment of the sigmoid sinus ipsilateral to the lesion. The thrombus in the venous sinus was aspirated under negative pressure to complete the clearance of the thrombus in the proximal segment of the venous sinus.
3. If the thrombus was located beyond the farthest location that the long sheath could reach, an intermediate catheter (Navien ™ 072, 115 cm Micro Therapeutics Inc. dba ev3 Neurovascular, USA or Sofia-plus 125 cm MicroVention Europe, France) was placed under the guidance of a PT2 microguide wire (0.014 × 300 cm Boston Scientific Corp., USA) and a Rebar 18 microcatheter (Micro Therapeutics Inc. DBA ev3 Neurovascular, USA) was delivered to the mesial segment of the superior sagittal sinus. Aspiration of the thrombus was continued using an intermediate catheter.
4. We artificially divided the superior sagittal sinus into 3 segments, with the anterior 1/3 referred to as distal, the middle 1/3 as mid, and the posterior 1/3 as proximal. The thrombus in the distal and mid segments of the superior sagittal sinus can be removed using intermediate catheter aspiration combined with a stent retriever. Under PT2 microguide wire guidance, a Rebar 18 microcatheter was delivered 1/3 anterior to the superior sagittal sinus to image and confirm thrombus location distantly. After the Solitaire FR (6 × 30 mm Micro Therapeutics Inc. dba ev3 Neurovascular) stent was delivered in place and released, thrombectomy was performed, which was conducted by distal and proximal segments.
5. After multiple stent retrievals, in patients still with significant vessel stenosis that affected venous blood return, a PTA balloon (6 × 30 mm Clearstream Technologies, Ltd., Ireland) was used to dilate the stenosis so that the vessel became patent.
6. In patients with general balloon dilatation effect, the indwelling microcatheter was used to pump tirofiban antiplatelet aggregation or unfractionated heparin anticoagulation.
7. In patients with more residual thrombus, thrombolysis with urokinase was administered intrasinusly (200,000 units).

## Results

### Baseline and Clinical Data of the Seven Patients

**Figure 1:**
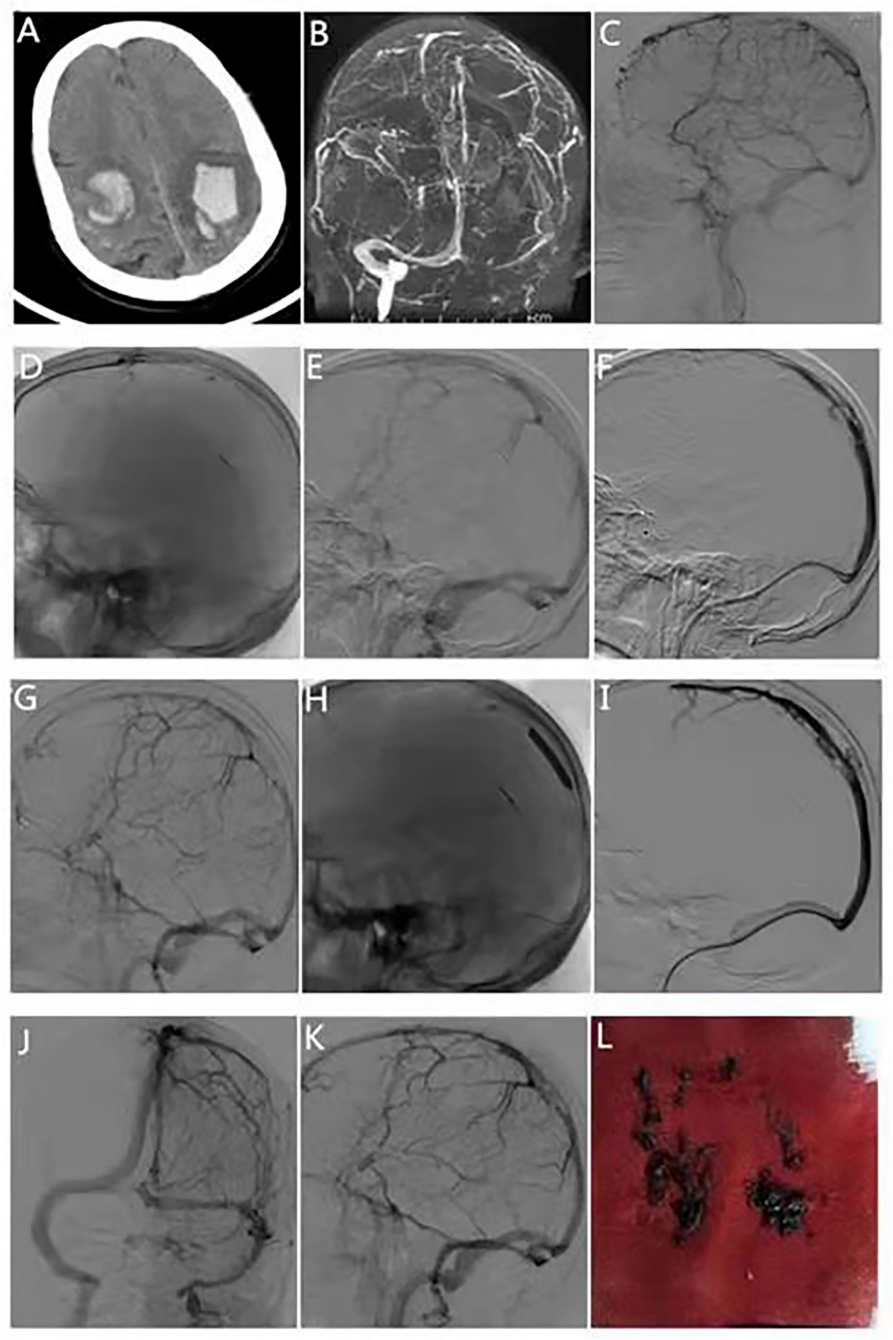
Typical case 1. The patient was a 66-year-old man, transferred from the outside hospital due to sudden unconsciousness, limb weakness, and episodic limb convulsions for 5 days. **(A)** Head computed tomography scan at the time of transfer shows bilateral parietal hemorrhage. **(B)** Magnetic resonance venography at the time of admission shows occlusion of the superior sagittal sinus. **(C)** Digital subtraction angiography shows occlusion of the middle and proximal sagittal sinus. **(D)** Microcatheter angiography suggests patency of the distal segment of the superior sagittal sinus. **(E)** Stent retrieval was performed for interruption of the superior sagittal sinus. **(F)** Mechanical thrombectomy of the middle segment of the superior sagittal sinus and delivery of the intermediate catheter into the proximal end of the superior sagittal sinus using the stent anchoring technique. **(G)** Residual stenosis in the middle segment of the superior sagittal sinus after thrombectomy of the middle segment and aspiration of the intermediate catheter. **(H)** Balloon dilatation of the middle segment of the superior sagittal sinus. **(I)** Intermediate catheter retrograde angiography suggests that the superior sagittal sinus is normal, stenosis is relieved. **(J,K)** Intermediate catheter retrograde angiography suggests that the superior sagittal, bilateral transverse, and sigmoid sinuses are well-visualized. **(L)** Venous thrombus has been removed.

**Figure 2:**
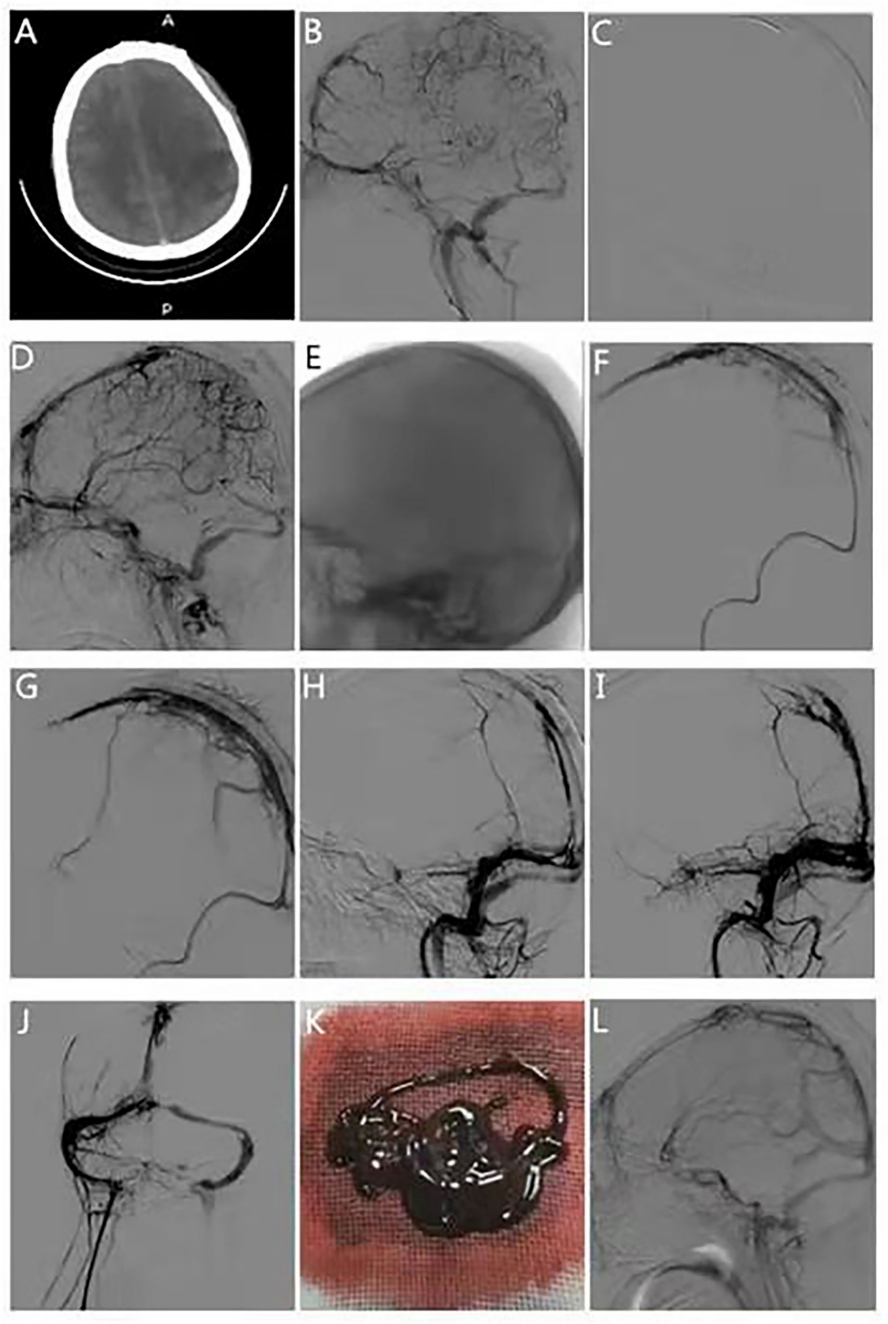
Typical case 2. The patient was a 29-year-old woman transferred from the outside hospital due to sudden headache, hemiparesis, coma, and limb twitching for 1 day. **(A)** Computed tomography scan of the head on admission suggests left parietal hemorrhage. **(B)** DSA suggests occlusion of the superior sagittal sinus, left transverse sinus, and sigmoid sinus. **(C)** Stent retrieval was performed from the distal part of the superior sagittal sinus. **(D)** The distal aspect of the superior sagittal sinus appears better than the anterior. **(E)** The intermediate catheter is delivered to the superior sagittal sinus midshaft using stent anchoring. **(F)** Thrombus retrieval in the middle segment of the superior sagittal sinus was performed using the solumbra technique. **(G)** The middle section of the superior sagittal sinus is better developed than before, but there is still proximal stenosis. **(H)** Stent retriever was performed to the proximal end of the superior sagittal sinus. **(I)** The posterior of the superior sagittal sinus appears better than the anterior. **(J)** Intravenous retrograde contrast suggests improved visualization of the superior sagittal, bilateral transverse, and sigmoid sinuses. **(K)** Intraoperative retrieved thrombus. **(L)** Review of DSA 90 days postoperatively suggests good visualization of the superior sagittal sinus.

We treated 7 patients with severe CVST (4 men, and 3 women). Procedures of typical cases are detailed in Figures 1, 2, respectively. The median age was 51 years (ranged from 15 to 66 years). The most common clinical symptoms were headache (85.71%) and focal neurological deficits (85.71%), followed by coma (57.14%) and epilepsy (42.86%). The following were etiologies: 1 patient with puerperium, 1 patient with essential thrombocythemia, 1 patient with oral contraceptive, 1 patient with protein S deficiency, 1 patient with dehydration, and 2 patients with an unknown cause. Imaging characteristics included the following features: 3 patients with cerebral hemorrhage, 3 patients with superior sagittal sinus hyperintensity signs, and 1 patient with cerebral infarction with intracerebral hemorrhage. The superior sagittal sinus was involved in 7 patients (100%), the transverse sinus in 5 (71.43%), and the sigmoid sinus in 4 (57.14%). Two patients had involvement of only the superior sagittal sinus was involved in 2 patients, and both the superior sagittal and transverse sinuses were in 1 patient, and the superior sagittal, transverse, and sigmoid sinuses were involved in 4 patients. The patient's basic conditions are shown in Table 1. After a diagnosis of CVST, all patients received low-molecular-weight heparin at a dose of 90–100 IU/kg/Q12 h. However, one patient rapidly developed hemiparesis and status epilepticus within 1 day during anticoagulant therapy. Thus, this patient received endovascular treatment on an urgent basis. The remaining six patients had persistently worsened or unrelenting neurological symptoms despite standard treatment. Thus, neuro-interventionalists and neuro-intensivists administered endovascular therapy to these patients after evaluating their conditions.

**Table 1:** Baseline characteristics of the seven patients.

**No**.	**Sex/Age**	**Presenting symptoms**	**Concurrent intracranial bleeding**	**Risk Factors**	**Location of CVT**	**Time between diagnosis and EVT, d**	**GCS score**
	**years**						
1	M/61	Headache, focal neurologic deficits	No	No identifiable	SSS/TS	9	13
2	M/55	Headache, coma	No	Dehydration	SSS/TS/SS	3	7
3	M/66	focal neurologic deficits, coma, seizure	Yes	No identifiable	SSS	6	5
4	F/29	Headache, focal neurologic deficits, seizure	Yes	Puerperium	SSS/TS/SS	1	12
5	F/51	Headache, focal neurologic deficits, seizure	Yes	Essential thrombocythemia	SSS	2	9
6	F/43	Headache, focal neurologic deficits	Yes	Contraceptive pills	SSS/TS/SS	6	15
7	M/15	Headache, focal neurologic deficits, coma	No	Protein S deficiency	SSS/TS/SS	2	8

*M, male; F, female; EVT, endovascular treatment; GCS, Glasgow Coma Scale (score range: 3–15, with the highest score indicating ordinary consciousness); CVT, cerebral venous thrombosis; TS, transverse sinus; SSS, superior sagittal sinus; SS, sigmoid sinus; StS, straight sinus*.

### Surgical Data of the Included Patients

The median time from onset to surgery was 3 days (range 1–9 days), and the median preoperative Glasgow Coma Scale was 8 (range 5–15). All 7 patients were treated with stent retriever therapy. Combined intermediate catheter aspiration was done in 4 patients, and balloon regional dilatation were done each in 4 patients, 2 patients were treated with unfractionated heparin regional anticoagulation therapy, 4 patients received tirofiban regional antiplatelet aggregation therapy, and 1 patient received urokinase 200,000 single site regional thrombolysis. The median number of stent retrievals was 5 (2–7 times), and the median duration of the procedure was 260 min (150–420 min) (Table 2).

**Table 2:** Clinical data of the seven patients.

**No**.	**Intermediate catheter**	**Type and size of balloon**	**Balloon expansion times**	**Type and size of stent retriever**	**Retrieval attempt**	**Intrasinus administration**	**Duration of intrasinus administration**
					**Times**		
1	No	PTA 5 × 30 mm	2	Solitaire FR,	2	Tirofiban 0.4 ug/kg/min	24 h
				6 × 30 mm			
2	No	No	0	Solitaire FR,	5	Urokinase 200 kU	0
				6 × 30 mm			
3	6F Navien	PTA 5 × 30 mm	3	Solitaire FR,	5	Heparin 500 U/h	24 h
				6 × 30 mm			
4	6F Navien	No	0	Solitaire FR,	7	heparin 500 U/h	24 h
				6 × 30 mm			
5	No	PTA 5 × 30 mm	2	Solitaire FR,	5	Tirofiban 0.4 ug/kg/min	24 h
				6 × 30 mm			
6	6F Navien	No	0	Solitaire FR,	3	No	0
				6 × 30 mm			
7	Sofia plus	PTA 5 × 30 mm	5	Solitaire FR,	6	No	0
				6 × 30 mm			

### Outcomes and Follow-Up of the Seven Patients

Four patients achieved complete recanalization (with uninterrupted flow in the venous system and ignoring some small residual thrombi attached to the sinus wall). Three patients achieved partial recanalization (interruption of continuous flow or narrowing of the lumen with more thrombi). No patient developed new cerebral hemorrhage or enlargement of the original hematoma after surgery. Furthermore, there was no occurrence of puncture site hematoma, vessel penetration, or other conditions. At the 90-day follow-up, 6 patients did not have any neurological deficits (mRS score 0), and 1 patient had left upper extremity paresis (mRS score 2) (Table 3).

**Table 3:** Treatment and follow up information of the seven patients.

**No**.	**Duration of operation**	**Recanalization degree**	**Post-treatment intracranial bleeding**	**MRs at last follow-up**
1	240 min	Complete	No	0
2	270 min	Complete	No	0
3	345 min	Partial	No	2
4	420 min	Partial	No	0
5	260 min	Partial	No	0
6	150 min	Complete	No	0
7	240 min	Complete	No	0

## Discussion

Anticoagulation is the first-line treatment for patients with CVST, but many patients experience persistent worsening symptoms after anticoagulation therapy (4). In this study, we evaluated 7 patients who had enormous thrombus burdens and superior sagittal sinus. Five patients had involvement of At least two venous sinuses were involved in 5 patients. Insensitivity may be related to a large thrombus burden. According to Liao et al. (16), patients with CVST who had poor outcomes and poor response to anticoagulant therapy had thrombi >10 cm in length, and involvement of the superior sagittal sinus was associated with a poor outcome. All 7 patients in our study achieved recanalization. Still, the rate of complete recanalization was only 57%. This result is lower than that in studies by Ilyas et al. (69%) (10) and Siddiqui et al. (74%) (11), and lower than that in the TO-ACT research (79%) (12). The lower rate of complete recanalization in our study may have been related to the large thrombus burden, with 71% of the patients having multiple venous sinus involvement.

The optimal endovascular therapy strategy for patients with CVST remains unclear, and current endovascular therapy for CVST still borrows endovascular therapy strategies for acute ischemic stroke (AIS) (13, 14). The 7 patients in this study were treated with stent retriever with a median of 5 retrievals (2–7). All 7 patients achieved a good outcome (mRS score 0 for 6 patients and MRS score 2 for 1 patient), which is quite different from the relationship between the number of retrievals for AIS and prognosis. The primary purpose of intravascular therapy for CVST is to recanalize venous vessels. Therefore, the operation time requirement is less than that required for arterial system diseases.

The median duration of surgery in this study was 260 min (range 150–420 min), which is significantly longer than that in the TO-ACT study (118 min; range 93–157 min) (12). This difference may have been related to the following factors: 1. In this study, all the venous pathways were transfemoral vein puncture, whereas in the TO-ACT study, most of the pathways were transjugular vein puncture, a condition that may shorten the operative time. 2. All patients in our study underwent dual-channel angiography, i.e., antegrade arteriography combined with retrograde venography, which is not described in the TO-ACT study. 3. Angiojet was the main endovascular therapy instrument in the TO-ACT study, and only five patients in that study were treated with thrombectomy stent. However, all patients in our study received thrombectomy. Because of a large thrombus load, the number of stent thrombectomy was greater than that in the TO-ACT study, and 4 patients were treated with large-bore intermediate catheter aspiration. Four patients were treated with a balloon, which increased the operative time.

Because of the high burden of venous thrombosis in our study, 4 patients were treated with combined intermediate catheter aspiration. With the excellent intermediate catheter compliance, some intermediate catheters could enter the proximal superior sagittal sinus. The Sofia plus catheter (17) and the 6F Navien catheter (18) have good compliance. The inner diameter of these catheters is larger than that of the ACE68 (Penumbra) catheter, and suction is stronger. We found that a large amount of thrombi could be aspirated with an intermediate catheter before stent thrombectomy, which helped improve the efficacy of endovascular therapy. Gorky et al. (19) applied the treatment to CVST with a large-bore suction catheter, and all patients achieved complete recanalization; further, 85% of patients achieved good prognosis. Sudeepta et al. (13) used a large-bore suction catheter to treat patients with CVST by stent anchoring. All patients achieved good recanalization without surgical complications. Most patients were discharged or entered the rehabilitation center. Sudeepta et al. (13) suggested that endovascular therapy with a large-bore suction catheter is a safe and effective treatment method for patients with CVST who have a poor anticoagulant effect. Because of the limitation of the length of the intermediate catheter, the thrombus in the middle and distal segment of the superior sagittal sinus needs to be removed with the stent, which is similar to the endovascular therapy strategy of large vessel occlusion in AIS. We found that all patients with stent thrombectomy combined with intermediate catheter aspiration had good prognosis. We believe that intermediate catheter aspiration combined with stent thrombectomy may be safe and effective for patients with CVST that had a large thrombus load.

A regional indwelling microcatheter was used for patients in this study who could not be recanalized completely after repeated thrombectomy in the middle and distal segment of the superior sagittal sinus. Wang et al. (14) adopted stent thrombectomy combined with long-term regional thrombolysis for patients with severe CVST for whom anticoagulation therapy was ineffective; the investigators achieved good results. Wang et al. (14) suggested that stent thrombectomy combined with regional thrombolysis was a safe and effective treatment option for patients with severe CVST.

### Limitation

This study has several limitations. First, it is a retrospective study, which limits the evidence levels of the results. Second, the data are from a single center. Multicenter studies are needed to expand the sample size and applicability of the conclusions. Third, given the small sample size of this study, a control group was not established in our analysis. Our study only reported the treatment of 7 patients and considering heterogeneity among patients, our findings remain to be confirmed by clinical trials with a larger sample size. In the future, we will conduct more strict randomized controlled trials to explore the optimal methods for the treatment of severe CVST.

## Conclusion

For patients with severe CVST who have an inadequate response to anticoagulant therapy, the prognosis of patients can be improved with a strategy of stent thrombectomy combined with intermediate catheter aspiration, balloon dilatation, regional thrombolysis or anticoagulation therapy to recanalize the occluded venous sinus quickly and effectively.

## Data Availability Statement

The original contributions presented in the study are included in the article/supplementary material, further inquiries can be directed to the corresponding author/s.

## Ethics Statement

The studies involving human participants were reviewed and approved by the Ethics Committee of the Third People's Hospital of Hubei Province (No. IEC-2021-16). Written informed consent to participate in this study was provided by the participants' legal guardian/next of kin.

## Author Contributions

TP: conceptualization, data curation, formal analysis, investigation, writing—original draft, and writing—review and editing. BD: conceptualization, data curation, formal analysis, funding acquisition, supervision, writing—original draft, and writing—review and editing. ZZ: data curation, formal analysis, and writing—original draft. BZ and JL: data curation, formal analysis, and writing—review and editing. All authors contributed to the article and approved the submitted version.

## Funding

This study was funded by Hubei Province Health Research Project (No. WJ2021F129) and Medical Research Project of Wuhan Municipal Health Commission (No. WX21B28).

## Conflict of Interest

The authors declare that the research was conducted in the absence of any commercial or financial relationships that could be construed as a potential conflict of interest.

## Publisher's Note

All claims expressed in this article are solely those of the authors and do not necessarily represent those of their affiliated organizations, or those of the publisher, the editors and the reviewers. Any product that may be evaluated in this article, or claim that may be made by its manufacturer, is not guaranteed or endorsed by the publisher.
